# Detection of Defects in Solid Carbide Cutting Tools During Creep-Feed Flute Grinding (CFG) Using Recurrence Analysis

**DOI:** 10.3390/ma18122743

**Published:** 2025-06-11

**Authors:** Marcin Sałata, Robert Babiarz, Krzysztof Kęcik

**Affiliations:** 1 Department of Manufacturing Techniques and Automation, Faculty of Mechanical Engineering and Aeronautics, Rzeszow University of Technology, 12 Al. Powstancow Warszawy Street, 35-959 Rzeszow, Poland; robertb@prz.edu.pl; 2 Department of Applied Mechanics, Faculty of Mechanical Engineering, Lublin University of Technology, 36 Nadbystrzycka Street, 20-618 Lublin, Poland; k.kecik@pollub.pl

**Keywords:** recurrence plots, recurrence quantification analysis, creep-feed grinding (CFG), flute grinding, cemented carbide, diamond grinding wheels

## Abstract

This study presents a comprehensive analysis of defect detection in the manufacturing process of solid carbide milling tools. The creep-feed flute grinding technique was used to fabricate a milling tool, with cutting force signals recorded and examined using recurrence analysis and conventional statistical methods. The analysis identified four distinct dynamic fluctuations (cutting force amplitude jumps), which showed a direct correlation with the formation of microcracks on the flute surface. These jumps exhibited varying levels of reduction, ranging from 5% to 22% in amplitude. A detailed investigation, including recurrence plots and recurrence quantification analysis (RQA) with a moving-window approach, revealed that several recurrence indicators, such as the recurrence rate (RR), determinism (DET), and maximum diagonal line length (L_MAX_), were highly effective in detecting microcracks, as their values significantly deviated from the reference level. These results were compared with conventional statistical analysis, and interestingly, the recurrence methods demonstrated greater sensitivity, successfully detecting additional very small cutting force jumps that conventional statistical methods could not identify.

## 1. Introduction

### 1.1. Characteristics of Creep-Feed Grinding

**Figure 1:**
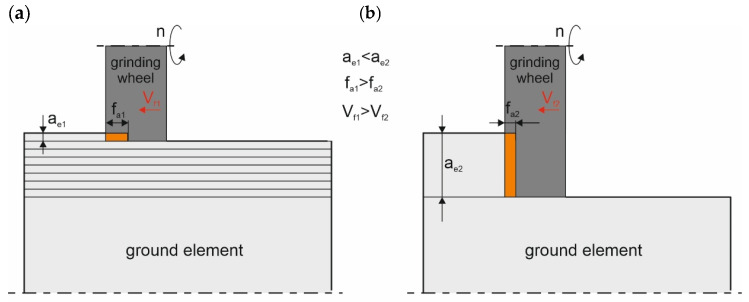
Grinding methods: (**a**) conventional grinding and (**b**) creep-feed grinding [9,18].

The primary challenges in the production of solid carbide tools include the manufacturing time, the complexity of tool geometry, and the elimination of production defects [1,2,3]. A main operation in carbide tool manufacturing is flute grinding, which accounts for approximately 70% of the total production time [2,4]. The flute formation process is multi-stage, requiring both rough and finish grinding, typically performed through multiple passes of the grinding wheel, significantly increasing the overall machining time [5,6]. To address these limitations, a novel grinding method named creep-feed grinding (CFG) has been developed [7]. This approach enables the removal of the entire machining allowance in a single pass of the grinding wheel, effectively replacing the conventional multi-stage rough and finish grinding processes simultaneously [8]. Deep Cut Grinding (DCG), commonly referred to in the literature as CFG, differs significantly from traditional grinding methods [9], as illustrated schematically in Figure 1.

In the CFG method, the workpiece feed rate (V_ft2_) is significantly lower compared to in conventional grinding. In the CFG process, material removal occurs in a single pass of the grinding wheel, with grinding depths (a_e2_) typically exceeding 1 mm [10,11]. In contrast, conventional grinding employs high feed rates (V_fa1_) and shallow grinding depths (a_e1_), requiring multiple successive passes to remove the entire material allowance [12,13]. As a result, the CFG process is widely used for machining difficult-to-machine materials, particularly in the aerospace and space industries, due to its high grinding efficiency [11,13]. Additionally, CFG is applied in the machining of cemented carbide tools and ceramics, especially in operations involving the formation of cutting tool flutes [11,12,13,14].

CFG is an advanced abrasive machining process characterized by its ability to remove substantial amounts of material in a single pass. This is achieved through a combination of a high depth of cut (usually from 1 to 10 mm) and a low workpiece feed rate (5–20 mm/min), resulting in a prolonged contact arc between the grinding wheel and the workpiece. This configuration enables efficient bulk material removal while maintaining tight dimensional tolerances and excellent surface finishes [14].

One of the most notable advantages of CFG is its high productivity in machining difficult-to-cut materials, such as hardened steels and superalloys, while simultaneously ensuring superior surface integrity without the need for secondary finishing operations. The low specific cutting forces help to reduce mechanical stress on both the tool and the workpiece, which can extend tool life and mitigate deformation risks. However, CFG also presents several limitations. The extended contact time and high material removal rates generate significant thermal loads, necessitating the use of high-pressure, precisely directed coolant systems to prevent thermal damage. Additionally, CFG requires specialized equipment with high dynamic and static stiffness, as well as robust spindles capable of handling elevated cutting forces. The process is also associated with increased grinding wheel wear and more frequent dressing cycles. Despite these challenges, CFG remains a highly effective solution for high-precision applications that demand both dimensional accuracy and superior surface quality.

Since the flute in solid carbide end mills is responsible for chip evacuation from the machining zone, its surface condition plays a critical role. Beyond roughness parameters, the presence of defects such as scratches or inclusions is also a key factor in assessing flute surface quality. In a study by the authors of [15], flute grinding in solid carbide end mills was investigated using a diamond grinding wheel with a 3.5 mm metal bond. The grinding process was carried out at speeds ranging from 15 to 40 m/s, with feed rates between 40 and 100 mm/min. The study found that these technological parameters had a statistically significant effect on the roughness parameter (Sa). However, due to the variability in the results, this effect remained minimal.

Another crucial factor in CFG that directly impacts both the grinding process and its outcomes is the selection of the appropriate grinding method and the corresponding coolant delivery technique. During the grinding of straight flutes in solid carbide end mills, with a flute depth of 4 mm, it was observed that the highest grinding force components (*Fn* = 250 N, *Ft* = 140 N) occurred with the climb grinding method, while lower force values (*Fn* = 210 N, *Ft* = 70 N) were recorded using the conventional grinding method [15]. However, when analyzing surface roughness, more favorable results were achieved with the climb grinding method. Similar trends were noted by Żyłka et al. [16], who compared climb and conventional CFG of Inconel alloys in surface grinding. Their study also found that climb grinding led to excessive heating of the workpiece, causing thermal damage to the ground surface. Nonetheless, the choice of grinding strategy did not influence the magnitude or type of residual stresses in the surface layer.

Among the various parameters of the grinding process, the grinding force has been the most commonly monitored variable in previously implemented supervision systems, as its behavior accurately reflects the characteristics of the process. Another key parameter for process monitoring is temperature measurement. High temperatures, particularly in single-pass grinding, can cause damage to both the ground end mills and diamond grinding wheels. Additionally, thermal expansion may lead to dimensional changes in the workpiece, resulting in geometric errors and defects such as cracks or grinding burns. Therefore, effective methods for detecting grinding defects are essential in the production of cutting tools. In reference to the above, Sałata [13] proposed a method for measuring the temperature during the grinding of flutes in solid carbide tools and conducted measurements of grinding force. Grinding burns were observed, enabling the identification and elimination of defective samples. Analysis of the recorded temperature signal from the grinding process revealed a direct correlation between temperature and the occurrence of burns. However, no such correlation was found when analyzing the grinding force signal. Additionally, it was demonstrated that the selection of an appropriate grinding wheel significantly influences the effectiveness and quality of the grinding process.

Analyzing machining methods for materials with difficult machinability, such as sintered carbide, Buk et al. [17] presented various techniques for shaping these materials in their study. The authors observed that while milling and grinding yield satisfactory results, they are hindered by rapid tool wear and complex geometries, making production and maintenance time-consuming. Erosion machining methods, including electrochemical machining and wire electrical discharge machining, show promising results, particularly for finishing operations, as they minimize tool wear and produce high-quality surfaces without defects. However, methods like these are still limited by surface layer defects, such as recast layers and microcracks, which require further investigation. Sułkowicz et al. [18] proposed a method to improve the shape and dimensional accuracy of low-stiffness shafts during single-pass grinding using a traverse grinding wheel. Their approach involves applying additional infeed based on the normal component of the grinding force. Experimental tests demonstrated that variable infeed reduced cylindricity deviations by 82% and dimensional errors by 90% compared to constant infeed. Despite an 11% increase in grinding force, surface roughness remained unaffected, with improved quality at both ends of the workpiece. This method provides an effective solution for grinding low-stiffness shafts without the need for steady rests.

Furthermore, it should be noted that any grinding process induces significant stresses in the workpiece, leading to both elastic and plastic deformation [19]. As a result, a force known as the cutting force acts on the grinding wheel face. This force increases the torque demand on the spindle, and is one of the fundamental parameters of the process. Although spindle torque is easier to measure, its slow variability—due to the spindle’s high moment of inertia—makes it less commonly used for cutting process monitoring. In contrast, cutting force measurement is widely employed for this purpose, as it is highly sensitive to tool condition [19,20].

The magnitude of the cutting force increases with tool wear and exhibits a sudden change when even a small portion of the blade chips or the workpiece material fractures [21]. In some cases, this change may be localized—if the blade retains its cutting properties after chipping, or if the grinding wheel moves past the damaged area of the workpiece. Such changes are more challenging to detect, and typically appear as small, sharp spikes in the signal [21]. Therefore, the cutting force measurement system must have a wide signal bandwidth to ensure precise detection. Strain gauges and piezoelectric dynamometers are commonly used for this purpose. The latter, in particular, are preferred due to their wide bandwidth and insensitivity to temperature variations. For this reason, the normal grinding force signal has been selected as the key measurement signal for detecting damage in the material being ground. However, a major drawback of this approach is its dependence on cutting parameters such as the depth of cut, cutting speed, and feed rate [22,23].

As a result, the raw normal force signal has limited usefulness as a diagnostic tool for detecting low-energy process state changes, such as micro-damage to the tool or microcracks in the workpiece. Additionally, self-excited vibrations occurring during grinding [24] often obscure signal variations caused by micro-damage. Due to the wide measurement bandwidth, these vibrations form a significant portion of the measured force signal. To mitigate their impact, low-pass filtering is applied. However, improperly chosen filtering parameters can further degrade the detection of microcracks.

Detecting microcracks during the tool grinding process requires balancing multiple, sometimes conflicting, factors. Given these challenges, it is necessary to identify indicators within the normal grinding force signal that can effectively detect short-term force spikes while remaining resistant to slower changes caused by grinding wheel wear or variations in machining allowance [25].

Recent studies have focused on advanced techniques for monitoring cutting tool conditions and detecting tool wear during operation. For instance, Oh and Lee proposed a model-based approach for tool wear detection and life prediction using force signals in end milling [26], while Hirsch and Friedrich developed a data-driven wear prediction method utilizing a single integrated sensor [27]. Other recent works explore tool–workpiece interactions under flank wear conditions [28], sensorless monitoring in broaching [29], and multimodal data fusion combined with deep learning techniques [30]. Although these methods provide valuable insights, they primarily address wear that occurs during tool usage (e.g., milling, drilling, or turning). In contrast, the present study focuses on a different issue—the detection of internal microdefects that arise during the manufacturing process of cutting tools.

Grinding research often highlights the importance of assessing grinding wheel wear, particularly when processing difficult-to-machine materials. Wang et al. [31] proposed an effective method integrating wavelet soft threshold noise reduction, Hilbert–Huang Transform (HHT), and a random forest (RF) algorithm to identify the wear state of a white corundum grinding wheel during GCr15 bearing steel grinding. Their approach involved decomposing acoustic emission (AE) signals using empirical mode decomposition (EMD), selecting relevant intrinsic mode functions (IMFs) based on correlation coefficients, and extracting key features—such as the maximum value, root mean square (RMS), and spectral centroid—from the selected IMFs. The grinding wheel wear condition was then categorized into three levels—mild, moderate, and severe—based on consistent results obtained for the wheel surface topography, workpiece surface roughness, and normal grinding force. This method achieved a classification accuracy of 93.3%, demonstrating its potential for real-time monitoring and optimization of dressing intervals. These findings highlight the growing trend in grinding research toward integrating signal processing and machine learning for intelligent wheel condition monitoring [5].

Tool wear during grinding directly affects dimensional accuracy and surface quality, making effective tool condition monitoring (TCM) critical in modern manufacturing. Pimenov et al. [32] reviewed both conventional and AI-based TCM systems, emphasizing the role of various sensors—including acoustic emission, vibration, force, temperature, and imaging—in detecting wear, predicting failures, and supporting maintenance decisions. Their findings underscore the importance of combining traditional sensing methods with AI-driven predictive models to enhance tool life management, process stability, and overall production efficiency.

### 1.2. Application of Recurrence Analysis

Recurrence analysis is based on the property of recurrence. It characterizes the behavior of nonlinear dynamic systems, including periodicity, stability, repetition, and chaos. Since the machining process is a highly nonlinear process, recurrence analysis is widely used in this field. Recurrence quantification has been applied to chatter detection [33] and tool wear monitoring [34,35].

Recurrence plot analysis has also been utilized for the detection of microcracks, cracks, and other forms of damage. In [36], the identification and localization of defects in fiber-reinforced plastic and carbon-fiber-reinforced plastic based on cutting forces were examined using recurrence quantification analysis. Two recurrence quantification methods were employed: one using a fixed threshold value and the other assuming a constant recurrence rate. The findings indicated that both methods were effective for defect detection. In [37], it was demonstrated that specific recurrence indicators could detect artificial defects. Furthermore, these indicators were validated on real damage scenarios, confirming their applicability. The influence of cutting parameters on defect detection and the sensitivity of recurrence plots were examined in [38]. This study highlighted the significant role of cutting parameters in defect detection, and revealed that certain indicators are affected by these parameters. Nicholas and Trickey [39] used multivariate RQA techniques to identify dynamic changes caused by cuts of varying lengths in a plate. Iwaniec [40] applied recurrence plot and quantification methods to analyze various mechanical systems. In [41], Iwaniec et al. selected four recurrence quantification measures to analyze the dynamic changes between three different harmonic excitation modes of an aluminum plate, as well as to compare cracked and uncracked metal plates. Qian et al. [42] used recurrence and entropy-based methods to develop a data-driven model for ball bearing degradation. Kecik et al. [43,44] evaluated the effectiveness of recurrence methods in detecting defects in angular contact ball bearings. Damages on various bearing components of different sizes were analyzed and successfully detected. The results demonstrated that recurrence quantifications, especially determinism and laminarity, were the most effective measures for detecting defects.

Additionally, the paper by Żyłka et al. [45] focused on the application of recurrence quantification analysis to evaluate the dynamics of toroidal milling processes. The analysis of cutting force components revealed that recurrence indicators, such as determinism, entropy, and the maximum value, are correlated with milling parameters like the feed per tooth and depth of cut. Notably, the axial force component exhibited distinct results, with recurrence indicators effectively capturing milling dynamics related to the cross-sectional area of the cutting layer. These findings suggest that recurrence indicators are valuable for analyzing the milling process in various directions and predicting tool wear. Furthermore, Lisowicz et al. [46] investigated tool wear during the turning of titanium alloys using recurrence plot and quantification methods. The results indicated that uncoated inserts had a longer tool life compared to coated ones. Recurrence quantification effectively detected changes in process dynamics, particularly with coated inserts, where tool wear and increased cutting forces were observed. Some recurrence quantification, along with recurrence graphs, helped to identify the critical wear point for coated tools. The study suggests that recurrence analysis can be used to assess machining dynamics, though further research is needed for uncoated tools. These findings underscore the efficacy of recurrence quantification techniques as powerful tools for structural health monitoring and defect detection.

### 1.3. Motivation and Aim

In summary, recurrence methods are extensively utilized in the study of cutting processes, particularly for detecting tool wear, assessing machining stability, and identifying chatter. The effectiveness of various recurrence indicators varies depending on the specific application. However, the correlation between recurrence indicators and defects, such as sample cracks, has yet to be systematically examined, despite its potential for analyzing cutting process dynamics. To the best of the authors’ knowledge, no publications exist that examine the dynamics of the flute grinding process with diamond grinding wheels using recurrence techniques. To address this gap, the present paper investigates the relevance of recurrence quantification analysis methods for detecting the first symptoms of defects based on the measured forces in the cutting tool grinding process.

The aim of this study is to analyze the dynamics of the grinding process of the tool and detect the first signs of breakage of the workpiece (or grinding wheel), manifested by the appearance of sudden peaks in the analyzed signal. The study identifies the most relevant recurrence measures for analyzing crack detection in ground carbide tools. The most significant recurrence indicators pertinent to the discussed process are highlighted, and classical statistical analysis results are provided for comparative purposes. Additionally, further statistical analyses are conducted.

## 2. Methodology of Experiment

**Figure 2:**
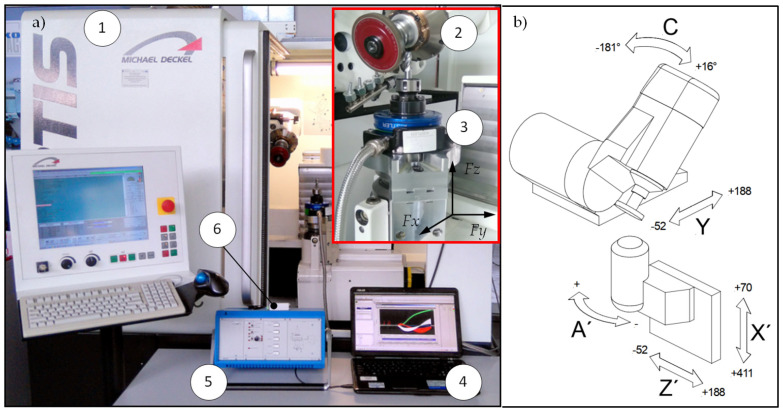
Stand for testing creep-feed flute grinding process. (**a**) Machine view: 1—five-axis grinding center; 2—grinding spindle with diamond wheel; 3—Kistler rotary dynamometer type 9123; 4—computer; 5—Kistler amplifier type 5223B; 6—A/C NI USB converter type 6009; *Fx*, *Fy*, *Fz*—dynamometer measurement directions. (**b**) Machine kinematics.

A dedicated test stand for investigating the flute grinding process of solid carbide end mills was developed based on the FORTIS five-axis grinding center (ISOG Technology GmbH, Weilheim, Germany) as a base (No. 1 in Figure 2a). Notably, this advanced tool grinder is designed for the production and regrinding of various cutting tools, including drills, milling cutters, taps, and custom-profile tools. It operates as a five-axis vertical CNC grinding center equipped with three linear axes (X, Y, Z) and two rotary axes (A, C), allowing full interpolation. Figure 2a presents a general view of the machine along with the measurement setup, while Figure 2b shows the machine tool axis configuration.

The testing stand includes a grinding spindle (No. 2 in Figure 2a), on which a diamond grinding wheel is mounted, and a dynamometer (No. 3 in Figure 2a), to which the workpieces are attached. The remaining devices (No. 4, 5, and 6 in Figure 2a) constitute the measurement system. Furthermore, the grinding forces were measured using a Kistler rotary dynamometer type 9123 (Kistler Group, Winterthur, Switzerland), a multi-channel piezoelectric device capable of recording the three components of grinding force, as well as torque, in two measurement ranges (no. 3 in Figure 2a). To enhance measurement accuracy and reduce noise, the system’s fine measurement range was selected, with the following limits: *Fx* = 0–450 N, *Fy* = 0–450 N, *Fz* = 0–1800 N. These values represent the upper bounds of the fine measurement range as specified by the manufacturer. In the present study, the recorded force values remained well below these limits.

**Figure 3:**
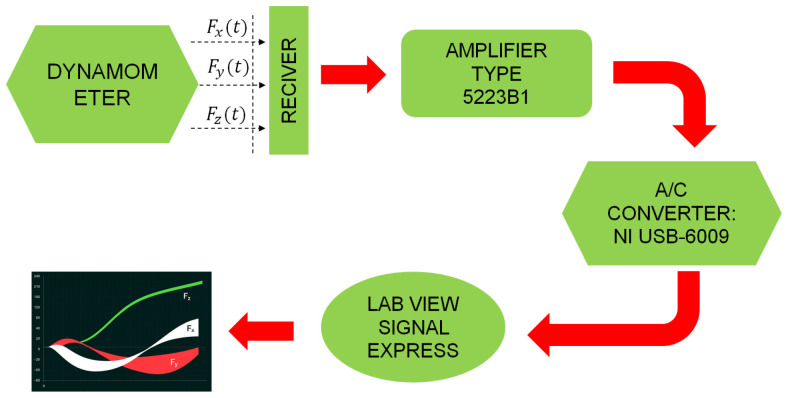
Schematic representation of developed measurement system.

The principle of the measurement system operation is graphically presented in Figure 3. The force components recorded by the rotary dynamometer are digitized via a 12-bit A/C converter and transmitted wirelessly to a stationary receiver. Subsequently, the data is relayed through a transmission cable to the 5223B1 signal amplifier (Kistler Group, Winterthur, Switzerland), where it is reconverted into an analog form for further processing. The signals are then fed into the NI 9215 A/C converter by National Instruments (Austin, TX, USA), controlled by LabView Signal Express 2013 V7.0 software, which records the measurement signals on a computer. The data acquisition rate of 5 kHz ensured sufficient resolution for measuring the grinding force components.

**Figure 4:**
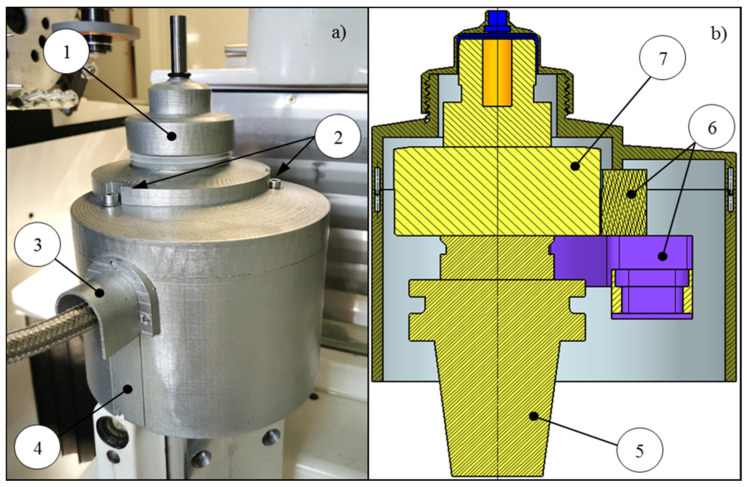
Dynamometer cover: (**a**) manufactured and installed cover; (**b**) cross-section area: 1—nut, 2—mounting screws, 3—protective cap, 4—slider, 5—SK50/HSK63 adapter, 6—mounting structure for stationary signal receiver, 7—dynamometer.

In addition to these measures, a custom dynamometer cover was designed and manufactured (Figure 4) to minimize the impact of the coolant on the accuracy of the grinding force measurements. The cover was produced using Fused Deposition Modeling (FDM), an additive manufacturing technique that ensures a precise geometric fit. Moreover, the printed cover was coated with a protective layer of paint, enhancing its resistance to coolant and environmental conditions. Its primary function was to shield the dynamometer from direct coolant exposure and prevent additional loads that could distort the measurement results. Additionally, the cover helped to protect the dynamometer from temperature fluctuations, which was crucial for maintaining data stability and accuracy.

The dynamometer (7) cover comprises two main components: a flange and a nut. The flange is firmly attached to the structure holding the stationary signal receiver (6) by screws (2), ensuring no physical contact between the cover and the dynamometer, allowing free rotation during grinding. The flange features a movable slider (4) for cable routing, while a protective cap (3) shields the cable connector from coolant exposure. The nut (1) is designed to provide quick and easy access to the tool clamping system, facilitating the replacement of carbide samples. The modular design of the cover allows for the accommodation of test samples with varying diameters. The SK50/HSK63 adapter (no. 5 in Figure 4b) allowed the connection of the dynamometer to the machine tool spindle interface.

## 3. Materials and Methods

### 3.1. Characteristics of Tool Grinding Process

**Figure 5:**
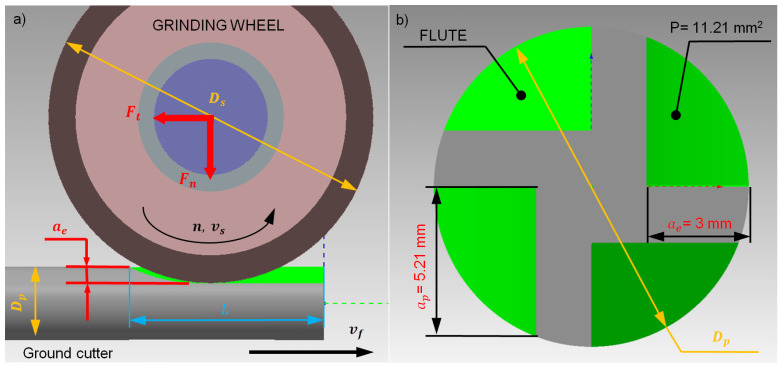
Kinematics of five-axis, single-pass flute grinding: (**a**) front view, (**b**) side view. *Ft*—tangential component of grinding force, *Fn*—normal component of grinding force, *D_s_*—grinding wheel diameter, *v_s_*—cutting speed, *v_f_*—feed rate, *D_p_*—blank diameter, *L*—grinding length, *a_p_*—grinding depth, *a_e_*—grinding width, *P*—cut layer cross-sectional area.

The research focused on the single-pass grinding process of straight flutes in solid carbide end mills made of ultrafine-grained cemented carbide (Figure 5). To eliminate the influence of process kinematics, the study concentrated on straight flute grinding, enabling a direct assessment of the grinding wheel’s characteristics.

In this single-pass grinding process, the grinding wheel operates at a constant grinding speed vs. and follows a straight trajectory at a feed rate *v_f_*. The grinding wheel, with a diameter *D_s_* and width *U*, penetrates the workpiece, which has a diameter *D_p_*, to a grinding width *a_e_* and depth *a_p_*. This way, the entire allowance, equal to the cross-sectional area *P*, is removed in a single pass, simultaneously creating a straight flute. The grinding width *a_e_* corresponds to the flute depth *a_p_*, while the grinding depth *a_p_* depends on the flute geometry and the diameter of the carbide rod *D_p_*.

The workpiece material comprised ultrafine-grained cemented carbide rods of type TSF22 (Ceratizit Group, Mamer, Luxembourg) with a diameter of 12 mm and a tolerance of h5. The material properties are presented in Table 1. The TSF22 grade exhibits exceptional hardness at 1930 HV and a transverse rupture strength of 4400 MPa, despite its low cobalt content (8.2%). The majority of carbide types possess a strength between 3000 and 4300 MPa. Considering the properties of the TSF22 carbide in comparison to all the sintered carbides produced, it can be classified among the most advanced tool materials. Among the available ultrafine grades, TSF22 demonstrates the most favorable balance between hardness and transverse rupture strength.

**Figure 6:**
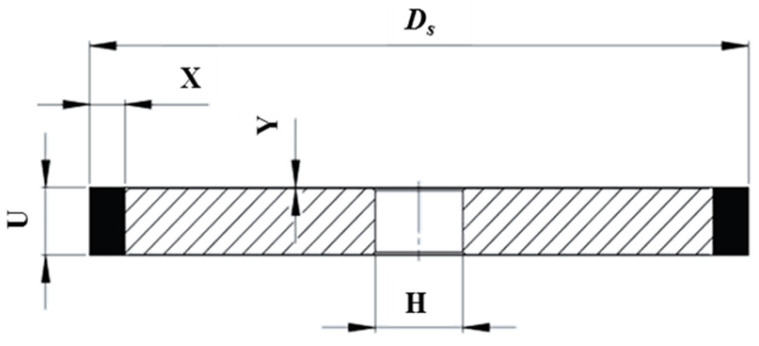
Geometric dimensions of type 1A1 grinding wheel: *Ds*—outer diameter, *U*—width, *X*—abrasive layer height, *H*—inner diameter, *Y*—chamfer size [13].

For flute grinding, type 1A1 diamond grinding wheels (Saint-Gobain Abrasives, Worcester, MA, USA) with a rectangular cross-section were utilized (Figure 6). Considering the diameter of the carbide rods and the flute geometry, grinding wheels with an outer diameter *Ds* = 100 mm, width *U* = 10 mm, abrasive layer height *X* = 10 mm, and inner diameter *H* = 20 mm were selected. The outer diameter was chosen to accommodate high grinding speeds while respecting the spindle speed limitations. The wheel width *U* was determined by the cross-sectional area of the cut layer, and the inner diameter was based on the grinding spindle holder. The abrasive layer height *X* was specified by the wheel manufacturer.

The experiments were conducted using the grinding parameters presented in Table 2. These parameters are typical for the manufacturing of tools using the CFG method.

### 3.2. Recurrence Analysis

The recurrence plot (RP) method is applied in various fields of engineering research. Recurrence plots were originally introduced as a visual tool for identifying various dynamics and detecting nonstationary behavior in time series data or nonlinear systems [47,48,49]. They are defined as square plots where both axes represent time, and recurrence states are indicated by dots at the corresponding positions. The RP is calculated using a recurrence matrix ***R_i,j_***:

(1) $$R_{i,j}=\left\{\begin{array}{cc} 1\; & if\;||x_{i}-x_{j}||\leq \varepsilon \\ 0 & otherwise \end{array}\right\},$$

where ***xi*** and ***xj*** are states at time *i* and *j*, *ε* is a threshold, and || || is a Euclidean distance.

Generally, the RP diagram is visualized as a binary image, where black dots represent ***R_i,j_*** = 1 (white space represents ***R_i,j_*** = 0). For a single scalar time series (experimental signal), the first step is to apply time-delay embedding in order to reconstruct the state-space trajectory (embedded signal) [50]. Then, the ***R_i,j_*** diagram is estimated from the embedded trajectory.

Analyzing the structure of RPs can reveal valuable insights into the underlying dynamics of a system. For example, diagonal lines in the RP, running parallel to the main diagonal, reflect the predictability and deterministic nature of the analyzed signal. Short diagonal lines can indicate transient dynamics, scattered points can suggest randomness and chaos, banding means intermittency, and white areas indicate sudden transitions or nonstationary behavior. The statistical analysis of recurrence structures has been expanded, with many recurrence indicators introduced. This method is collectively referred to as recurrence quantification analysis (RQA). These parameters assess the lengths of diagonal and vertical line segments within the recurrence patterns. The most commonly used quantifications include the recurrence rate (RR), determinism (DET), laminarity (LAM), entropy (ENT), trapping time (TT), recurrence times (T1, T2), average diagonal line lengths (L), maximum diagonal and vertical line lengths (L_MAX_, V_MAX_), recurrence period density entropy (RPDE), and clustering coefficient (CC).

The RR represents the proportion of recurrence points (black dots) in the RP diagram relative to the total number of points. A high RR value indicates regular behavior.

DET quantifies the percentage of recurrence points that form diagonal line structures, with a low DET suggesting less predictable behavior. In contrast, LAM measures the density of vertical line structures, providing insight into the system’s intermittency and laminar state. A low LAM value means more irregular behavior. Entropy (ENT) gauges the complexity of the RP diagram by analyzing the distribution of diagonal line lengths. A high ENT value signifies greater complexity or randomness within the system. The next recurrence quantification, L, characterizes the average length of diagonal lines. Longer diagonal lines reflect periodic or quasiperiodic behavior. A high L_MAX_ suggests periodic behavior or a prolonged period of predictability. V_MAX_ is useful for detecting intermittent behavior, with a low value indicating more frequent transitions between states, suggesting increased irregularity. The TT, indicated by vertical lines, measures the duration for which the system remains in a certain state. The quantifications T1 and T2 represent the percentages of horizontal and vertical lines, respectively. These parameters help to provide an understanding of the system’s stability, intermittency, and predictability. The RPDE indicator focuses on the deterministic structures within the recurrence plot. A low RPDE suggests that the system is more deterministic and predictable. Finally, the CC refers to the local density of recurrence points. A high CC indicates that the system tends to return to similar states regularly, suggesting periodic or quasiperiodic behavior, while a low CC value points to unpredictable dynamics. A detailed explanation and mathematical definition of these recurrence quantifications can be found in many papers [43,44].

## 4. Results and Analysis

### 4.1. Measured Grinding Force and Defect Identification

**Figure 7:**
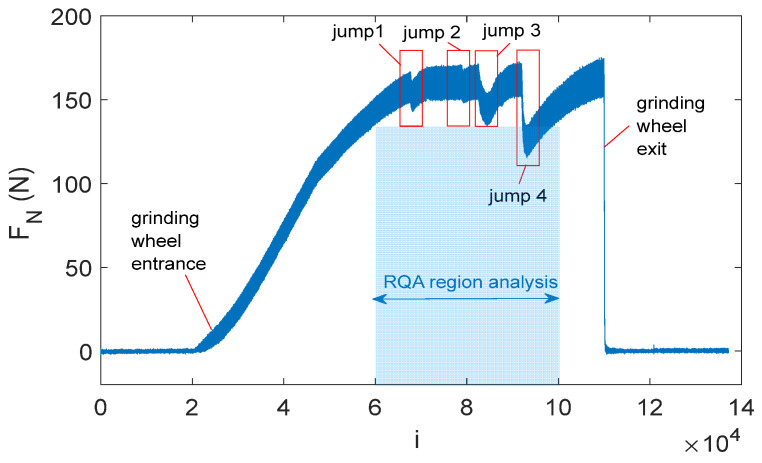
Time series of normal force of grinding process with parameters *v_c_* = 40 m/s and *v_f_* = 100 mm/min.

The first outcome of the study was the measurement of cutting forces recorded during the creep-feed flute grinding. Figure 7 shows the time series of the feed force, with several characteristic regions. Following the grinding tests, a statistical analysis of the cutting force components (Section 4.2) and a recurrence analysis (Section 4.3 and Section 4.4) were subsequently conducted.

Analyzing Figure 7, it can be observed that the point where the grinding wheel enters the material occurs around the 25,000th data point, while the completion of the grinding process is observed at approximately the 110,000th data point. The grinding process stabilizes near the 72,000th data point, where the force amplitude reaches a consistent value.

Analyzing the proper machining region (marked by the light-blue box), four distinct jump regions can be observed, highlighted by red boxes. In these regions, the amplitude of forces exhibits sudden jumps. In regions 1 and 2, the force stabilizes at a level similar to that before the jumps. However, in regions 3 and 4, the jumps are significantly more intensive.

**Figure 8:**
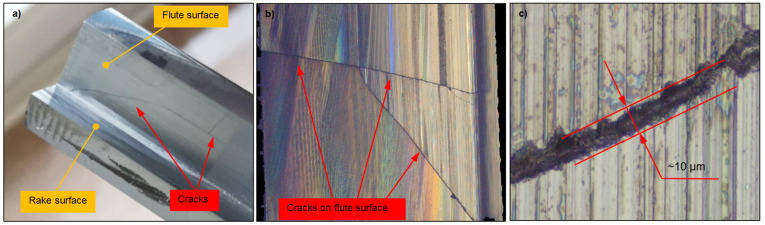
Surface topography images of end mill flute with visible cracks, acquired using Alicona Infinite Focus microscope: (**a**) actual image, (**b**) surface topography obtained with 2.5× objective, and (**c**) surface topography obtained with 100× objective.

In the analyzed case, the recorded force spikes during the grinding process (jumps 1–4) were directly responsible for the formation of the cracks shown in Figure 8. These defects were identified on the flute surface, the rake face, and the cylindrical surface. Additionally, cracks were observed in both the upper and lower sections of the end mill. Figure 8 highlights selected cracks that were detectable under appropriate lighting conditions without the need for additional equipment (Figure 8a). Despite the presence of multiple defects, the examined sample initially appeared intact. Only microscopic analysis revealed cracks that remained undetectable under standard visual inspection (Figure 8a,b). Using a 100× magnification lens, the width of the resulting cracks was measured, averaging 10 μm. Additionally, it was observed that the cracks exhibited an irregular edge (Figure 8c). The analysis of the obtained images revealed that the cracks were oriented in various directions, including both axial and radial, regardless of the grinding direction (Figure 8b).

Defects that occur during the manufacturing process of cutting tools are often undetectable, primarily because quality control focuses on geometric measurements rather than detailed surface analysis for micro-damage. The absence of dedicated monitoring systems and the use of coolant further complicate defect detection, as illustrated in Figure 8. Unfortunately, only catastrophic cracks, which are immediately visible, are typically addressed during production. It is important to note that non-destructive testing methods, such as Fluorescent Penetrant Inspection, could be highly effective in identifying microcracks. However, these techniques are not commonly employed in the quality control of cutting tools. Additionally, in the later stages of production, protective coatings are applied to the tool surface, further masking existing damage and making detection more challenging. Cracks and microcracks significantly affect cutting tool performance in milling, shortening tool life and increasing the risk of premature failure. Early detection during manufacturing is therefore essential for effective quality control.

### 4.2. Statistical Analysis Results

**Figure 9:**
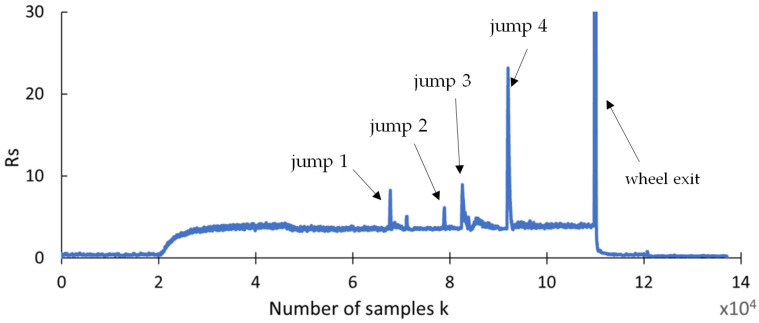
Range indicator *Rs*.

**Figure 10:**
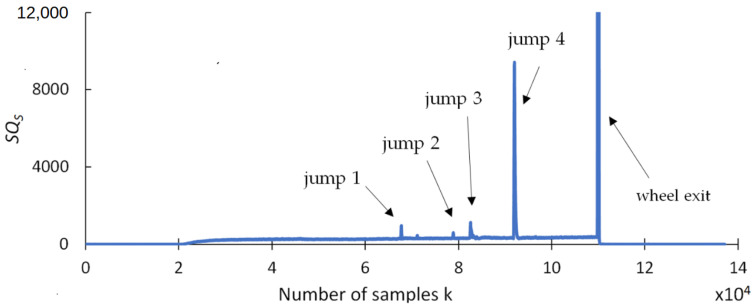
Square deviation, *SQ_S_*.

**Figure 11:**
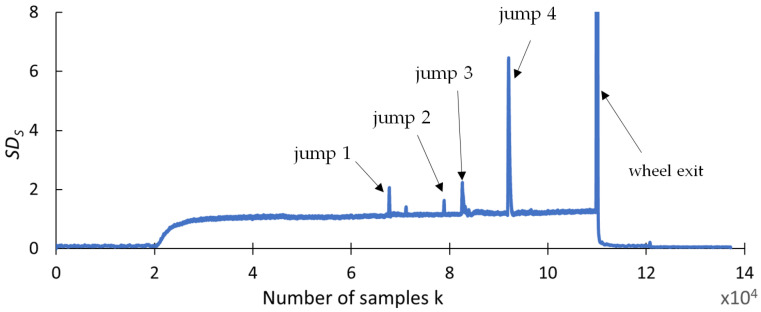
Standard deviation, *SD_S_*.

**Figure 12:**
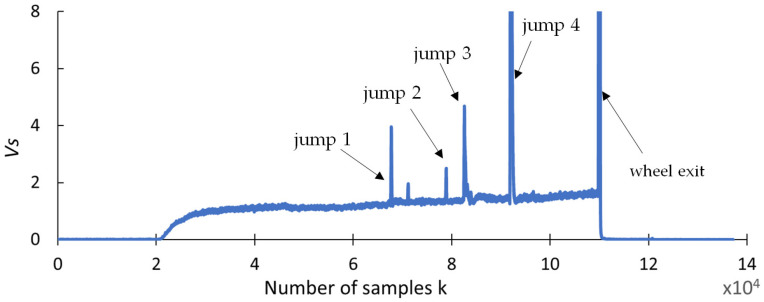
Variance V_S_.

**Figure 13:**
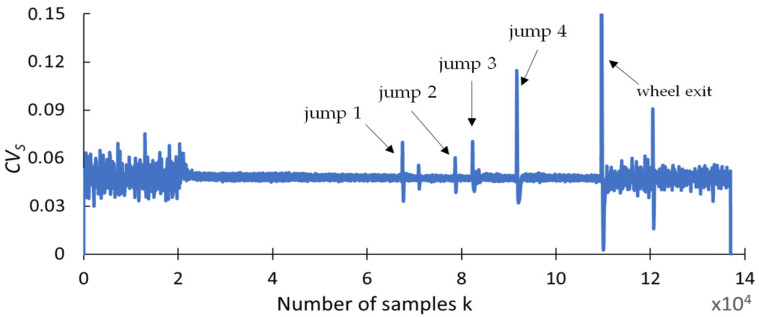
Coefficient of variation *CV_S_*.

**Figure 14:**
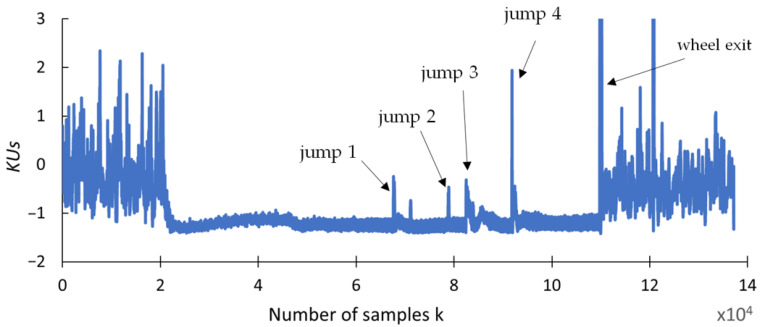
Kurtosis *KU_S_*.

**Figure 15:**
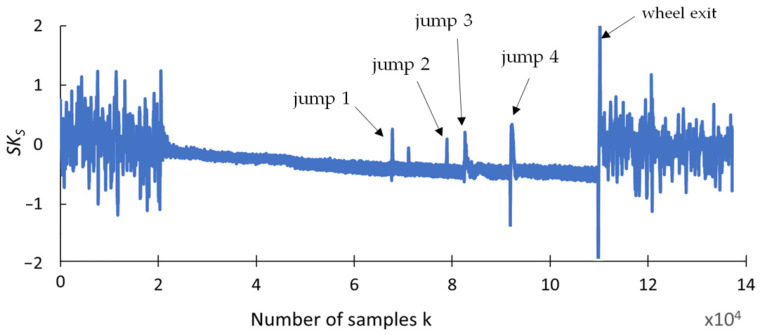
Skewness *SK_S_*.

This section presents the findings of research conducted to determine whether any changes occurred in selected statistical indicators due to the presence of cracks formed during the grinding of the carbide rod. The aim of this analysis was to provide a preliminary statistical characterization of the grinding force signals prior to recurrence analysis, in order to explore potential correlations between the data distribution properties and the presence of tool defects. An effective indicator should be highly resistant to interference and should provide a clear response when discontinuities appear in the grinding force signal. The statistical parameters used for this purpose include the range (R_S_), square deviation (SQ_S_), standard deviation (SD_S_), variance (V_S_), coefficient of variation (CV_S_), kurtosis (KU_S_), and skewness (SK_S_), and are presented in Figure 9, Figure 10, Figure 11, Figure 12, Figure 13, Figure 14 and Figure 15. Statistical analysis was performed using Statistica 13.3 software (TIBCO Software Inc., Palo Alto, CA, USA).

An analysis of the chart in Figure 9 reveals that the range indicator (*Rs*) exhibits significant variations in magnitude at the points in the waveform where signal spikes occur. Furthermore, for a monotonic waveform, the values remain stable, except at the locations influenced by these spikes. This suggests that *Rs* can be used both for detecting jumps and as a defect indicator.

Next, Figure 10 illustrates significant variation in the amplitude of the *SQs* index. The square deviation index *SQs*, due to its inherent characteristics, disproportionately emphasizes larger spike values, while smaller changes in the signal are less noticeable. This is an undesirable trait, as the ability to detect as many microcracks as possible is a key requirement. The *SQs* can detect four jumps and can be used for crack identification.

The waveform of the standard deviation index *SDs*, shown in Figure 11, like the Rs spread signal, clearly changes values with spikes in the strength signal and maintains the correct ratio between signal values. This makes it possible to unambiguously detect smaller signal spikes than in the *SQs* signal.

When analyzing the variance signal vs. shown in Figure 12, it can be seen that its course is similar to that of the square deviation index *SDs*. The index excessively differentiates the error values. Against the background of large values, jumps with smaller values appear less pronounced.

Analyzing the coefficient of variation index *CVs* shown in Figure 13, it can be seen that its course is similar to that of the *SD* square deviation index. The index excessively differentiates the error values. Against the background of large values, jumps with smaller values are less pronounced.

The waveforms of the last two selected indicators are summarized in Figure 14 and Figure 15. The analysis of their characteristics indicates that the indicators of kurtosis *KUs* and skewness *SKs* of the course of normal grinding force have characteristics similar to that of the variance indicator Vs. However, the changes in the values of these indicators exhibit less dynamicity than the variance and less stable waveforms at points of monotonicity in the course of grinding force.

**Figure 16:**
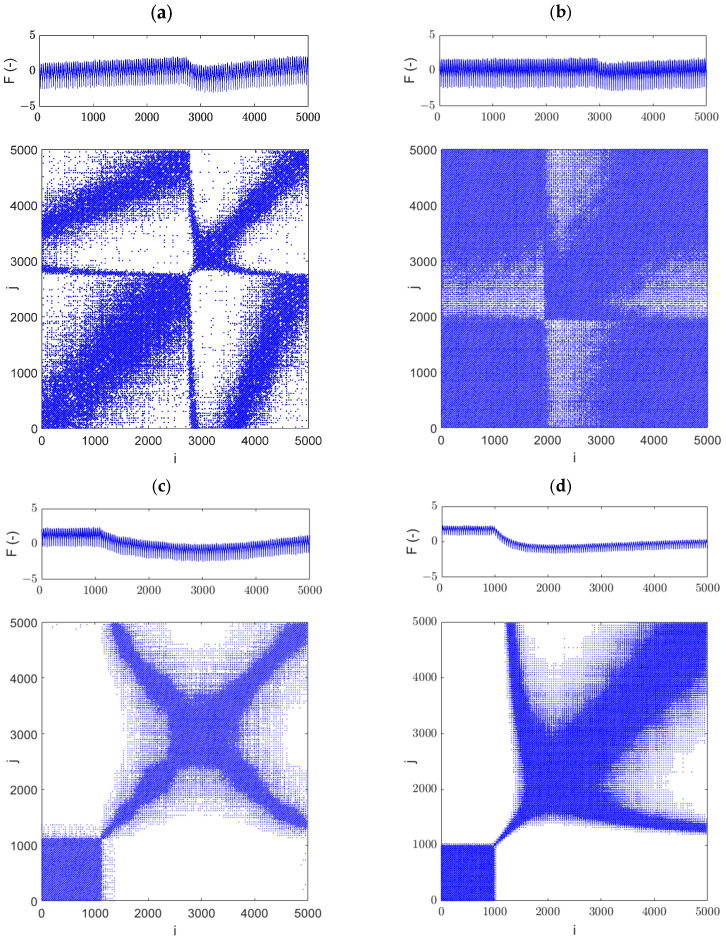
Recurrence diagrams obtained from the regions of force jumps in the grinding process: jump 1 (**a**), jump 2 (**b**), jump 3 (**c**), and jump 4 (**d**). The embedding parameters were set to *m* = 5, *d* = 5, with a threshold of ϵ = 0.4.

To facilitate comparison of the statistical indicators, they were normalized across three levels. Figure 16 illustrates the amplitudes of the analyzed statistical indicators for three selected signal level jumps (j1, j2, j3). The values have been standardized, with the highest amplitude jump (j4) set to 1.

### 4.3. Recurrence Plot Results

Due to the high number of data points, which reduces the readability of the recurrence diagrams, separate RPs were generated for each of the four jumps (no. 1, 2, 3, and 4). Above each RP diagram, the grinding force signal, normalized to a zero mean and a standard deviation of one, is displayed. The embedding parameters were estimated using the average mutual information (MI) and false nearest neighbors (FNN) algorithms. For all cases, the embedding parameters of time lag (d) and embedding dimension (m) were equal to 5. The threshold was assumed to be 0.4.

The RP for jump area 1, calculated from 5000 data points, is presented in Figure 16a. It can be observed that between approximately 2800 and 3100 data points, the plot differs noticeably, containing fewer recurrence points. Following this, the recurrence plot exhibits a structure similar to that observed prior to the first jump. In Figure 16b, the RP for the second jump is presented. Between approximately 2000 and 3000 data points, a white cross is visible, indicating sudden dynamic changes in the analyzed signal. Interestingly, in the time series (top panel), the amplitude jump becomes noticeable only at around 2900 data points. This suggests that RPs are more sensitive to such changes.

Comparing both recurrence diagrams (Figure 16a,b), it can be observed that they have different structures. This is caused by the fact that the amplitude before and after the jumps is different (Figure 16a) and the signal is not stabilized. However, in Figure 16b the force jump is small and does not significantly influence the amplitude. This means that jump no. 2 is less intensive than jump no. 1. Analysis of the RP diagrams reveals that jumps no. 3 (Figure 16c) and no. 4 (Figure 16d) are significantly more intense than jumps no. 1 and 2 (as also seen in the normalized time series—see the top panels). The RP for jump no. 4 contains the fewest recurrence points and a large white region, indicating that this jump is extremely strong.

In summary, the white areas indicate the presence of sudden dynamic changes in the analyzed signal. The larger these white areas, the more abrupt the changes. From this, we can conclude that if a fracture occurs during tool grinding, it generates an amplitude force jump, which can be detected by the RP method.

### 4.4. Recurrence Quantyfication Results

RQA is a method used to analyze the structures and recurrence plots through statistical analysis. It helps to estimate the dynamic characteristics of a system, such as its stability, periodicity, and chaotic behavior. In our RQA, the moving-window technique (MWT) was employed. This technique divides the time series data into smaller, overlapping segments of a specified size (window size). The window moves step by step (step size) across the entire time series. It offers improved temporal resolution compared to analyzing the whole time series at once, especially for systems with complex dynamics. This method calculates the local dynamics of recurrence quantification, and is useful for a detailed examination of dynamic changes within the time series.

**Figure 17:**
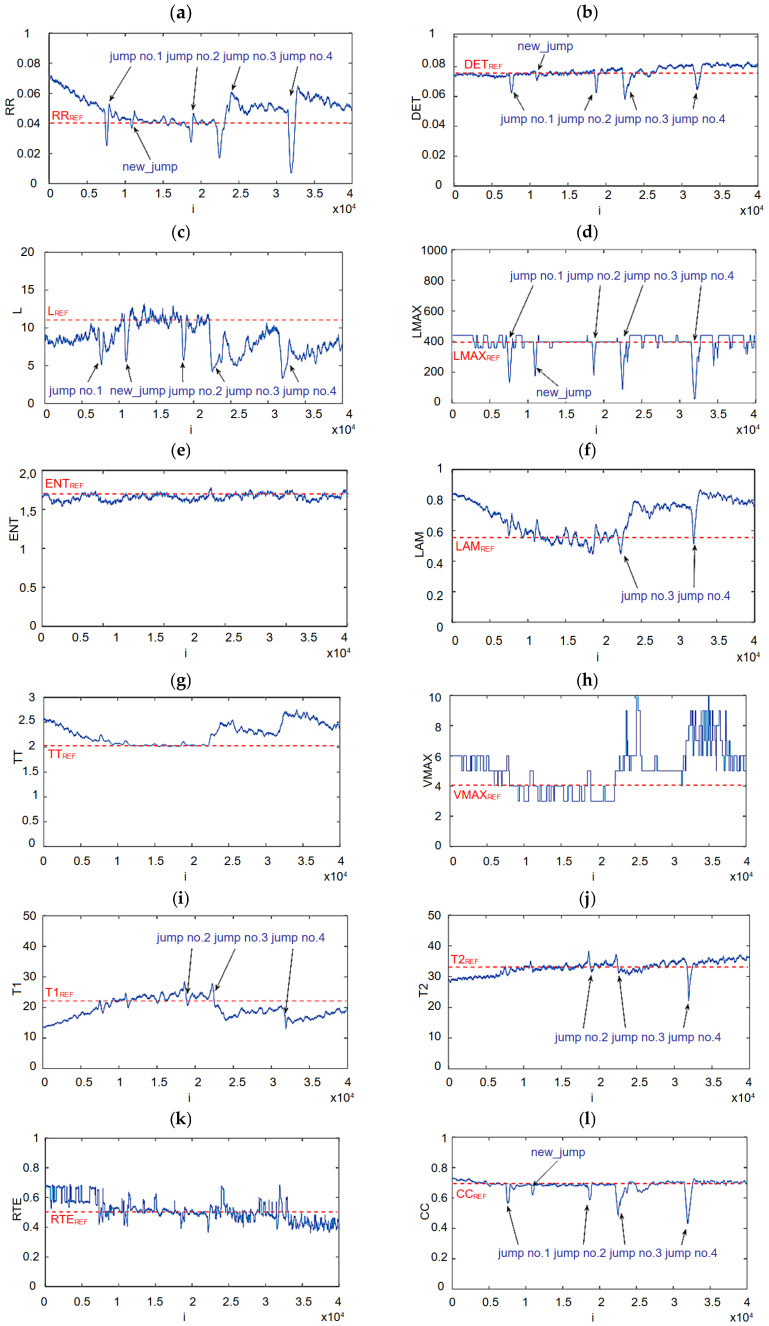
The results of the MWT method with a window size of 500 data points: RR (**a**), DET (**b**), L (**c**), L_MAX_ (**d**), ENT (**e**), LAM (**f**), TT (**g**), V_MAX_ (**h**), T1 (**i**), T2 (**j**), RTE (**k**), and CC (**l**). The embedding parameters were set to *m* = 5, *d* = 5, with a threshold of ϵ = 0.4. The window size was set to 500 points, and the window step to 1.

In Figure 17a, the results of the MWT are presented. The data points selected for analysis range from 60,000 to 100,500 (a slightly larger region than the blue region shown in Figure 7). The window size was set to 500 points, and it moved by one point at a time (window step = 1). This means the selected time series region was divided into segments, each containing 500 consecutive data points. After the first window (covering data points 1 to 500), the window shifted forward by one data point, so the next segment covered data points 2 to 501. The window moved by a small step, resulting in each new segment overlapping with the previous one by 499 data points. Each window was then used to perform the RQA. The four-jump region was identified in the time series analyzed using the MWT. Jump no. 1 occurs at approximately 8000 data points, jump no. 2 at around 19,000 data points, jump no. 3 at 22,000 data points, and jump no. 4 near 32,000 data points.

The reference RR index, which represents the percentage of recurrence points in the stable region, is equal to 0.04 (RR_REF_ = 0.04). This reference level was estimated based on portion of the signal without jumps. In the jump regions, a sharp temporary drop in the RR value (strong peaks) is observed. Naturally, the higher the jump, the more pronounced the corresponding peak. This indicates that the RR parameter can be used as an indicator of microcracks. Interestingly, at approximately 11,000 data points, small peaks are observed that are not reflected as amplitude jumps in the time series. This may suggest the presence of subtle dynamic changes that are not directly visible in the time series. Additionally, it is worth noting that a decrease in the RR is observed in the early part of the sequence. This is caused by an increase in force amplitude, which has not yet stabilized. Furthermore, after jumps no. 3 and no. 4, the RR value is significantly higher than the reference level, which could also be useful for diagnostics.

Similar conclusions can be drawn for the DET index (Figure 17b). Its value drops sharply at the locations of the peaks. The reference value, DET_REF_, is approximately 0.75. The temporary DET value after the third jump does not return to the reference level, but remains elevated. An additional peak is also noticeable when analyzing this recurrence index.

The analysis of the L index also allows for the detection of all four peaks, as well as an additional peak that is quite clearly visible (Figure 17c). The reference value is at the level of L_REF_ = 11 and is achieved in the region of stabilized force amplitude (10,000–12,200). The L_MAX_ value oscillates around the reference level of 400 (Figure 17d). This index also enables the detection of all five peaks. For larger force jumps, these oscillations become more pronounced, and a significant reduction in peak values occurs. A lower value of this index at the peak locations indicates that the motion is less periodic.

As for the entropy (Figure 17e), we observe that its value throughout the range is close to the reference value (ENT_REF_ = 1.7). Even at the peak locations, no noticeable ENT change is observed. We can conclude that this index is not very sensitive to amplitude jumps. Analyzing the recurrence index LAM (Figure 17f), it can be observed that only two strong peaks are clearly visible (peaks no. 3 and 4). Smaller jumps have values close to the reference level (LAM_REF_ = 0.55). It is worth noting that this index behaves very similarly to the RR index (Figure 17a).

The TT index also does not show changes at the peak locations (Figure 17g). However, based on this index, we can estimate whether the signal has stabilized. When the instantaneous TT value deviates from the reference level (TT_REF_ = 2.05), we observe that the signal has not yet stabilized. Similar conclusions can be drawn from the analysis of the V_MAX_ index (Figure 17h). The T1 index (Figure 17i) and T2 index (Figure 17j) behave similarly, with a noticeable difference for larger peaks. Smaller peaks are very difficult to detect using these indices.

The RTE index (Figure 17k) changes its value at a similar level across the entire analyzed range, making it difficult to detect peaks using this index. The last recurrence indicator maintains a fairly steady level around the reference value (CC_REF_ = 0.7, Figure 17l). Only at the peak locations does its value significantly drop. Therefore, it is one of the more promising indicators for detecting sudden changes in dynamics.

To summarize, we selected five RQA parameters—RR, DET, L, L_MAX_, and CC—that are capable of detecting microcracks during the production of a cutter using the creep-feed grinding process. In comparison, the recurrence indicators used for detecting tool wear and defects in composite materials based on cutting force have included the following: RR, DET, LAM, TT, ENT, and L_MAX_ [34]; L, ENT, V_MAX_, and T2 [36]; LAM, DET, TRANS, L, ENTR, RPDE, CC, TT, T1, and T2 [37]; and RR, DET, L, ENT, T1, and T2 [38]. As we can see, the choice of recurrence quantification indicators depends on the specific process, making it challenging to select a set of universal recurrence indicators. Furthermore, only four indicators were chosen for detecting microcracks in the grinding process, suggesting that this method is particularly difficult to diagnose.

## 5. Conclusions

This paper presents research on the detection of microdefects in cutters manufactured through the grinding process, utilizing measured cutting forces. This issue is particularly critical in industrial applications, as microcracks often go unnoticed when the tool meets dimensional tolerances. This approach is distinguished by its novelty in the field, as it is applied during the initial stage of cutter production.

The analysis of cutting force measurements revealed four distinct amplitude jumps at varying levels. The first two jumps exhibit similar characteristics, with an amplitude force of approximately 156 N and a relatively minor variation of about 5%. However, the third jump shows a decrease of approximately 10%, while the fourth drops by 22% compared to the stable grinding process. These jumps are considered the initial indicators of defects emerging in the ground tool. Therefore, the regions with jumps were analyzed in detail using a classical approach of statistical analysis (range, square deviation, standard deviation, variance, coefficient of variation, kurtosis, and skewness), and using recurrence analysis, which is more advanced method dedicated to nonlinear signals.

The statistical analysis revealed that the most effective indicators for microcrack detection are the standard deviation and the coefficient of variation. Among the evaluated statistical indices, *SDs* and *CVs* were ultimately selected as the most suitable, as they accurately reflect signal spikes across both low and high amplitude ranges. Additionally, these indicators demonstrate stable values during the process and relatively low values before and after the onset of machining, making them reliable for diagnostic purposes. For instance, in the case of jump 1, the value of the RS indicator increased by approximately 100%, SDs by 80%, vs. by 140%, and CVs by 40% compared to the reference values obtained in the stable phase of the grinding process.

The recurrence plot revealed distinct structural changes, indicating that this jump led to modifications in recurrence patterns and a significant reduction in the number of recurrence points. Additionally, the moving-window technique used in the recurrence quantification analysis enabled the determination of a reference level for recurrence quantification. This method also identified an additional jump, which may indicate the presence of a very small microcrack. Notably, this new jump was not detected through statistical analysis. The most effective recurrence quantification measures were found to be the recurrence rate, determinism, average diagonal line length, maximum diagonal line length, and clustering coefficient. These indicators successfully detected all the analyzed microcracks, with their values deviating significantly from the reference level. Moreover, these indicators enabled the detection of additional jumps of very small amplitude.

The next step of our analysis will involve developing a universal recurrence indicator based on selected quantifications, which can be implemented in a grinding machine to enable the direct detection of microcracks during the manufacturing process. Additionally, various machining parameters, such as the feed rate, depth of cut, and cutting speed, will also be considered in our approach.

The proposed method for defect detection during the manufacturing of cutting tools has demonstrated high effectiveness under laboratory conditions. However, its application in industrial environments presents several significant limitations. First, the use of a force sensor as an additional adapter in the tool clamping system requires a reduction in the maximum overhang of the ground tools. Moreover, it necessitates modifications to the machine enclosure to accommodate wiring, the installation of a signal receiver, and additional mounting components. The presence of the force sensor also affects the coolant delivery—its direction must be adjusted to avoid direct impact on the workpiece, which may reduce cooling efficiency. Additionally, the need for a protective cover to shield the measurement system from coolant impacts complicates the assembly and disassembly of the setup and negatively affects process ergonomics.

Future studies should focus on adapting the proposed diagnostic method to industrial conditions, including the integration of wireless force measurement systems and non-invasive sensor-mounting techniques. Additionally, further research should investigate replacing force signal analysis with other diagnostic signals, such as acoustic emission or vibration, to enhance the robustness and reliability of defect detection in real-world manufacturing environments.

## Figures and Tables

**Table 1:** Properties of ground carbide.

Grade	Classification	Grain Size [μm]	Co [%]	Density [g/cm^3^]	Hardness HV30	Transverse Rupture Strength [MPa]
TSF22	Ultrafine	0.2–0.5	8.2	14.5	1930	4400

**Table 2:** Grinding parameters.

Workpiece Material	Cutting Speed [m/s]	Feed Rate [mm/min]	Grinding Direction	Grinding Length [mm]	Grinding Depth [mm]	Grinding Width [mm]	Coolant
TSF22	40	100	Down grinding	30	5.21	3	Grinding oil

## Data Availability

The original contributions presented in this study are included in the article. Further inquiries can be directed to the corresponding author.
